# Superconductivity in ordered Li–Al–B compounds

**DOI:** 10.1038/s41598-024-84542-6

**Published:** 2025-01-02

**Authors:** K. Hussain, S. J. Donaldson, E. Karaca, P. J. P. Byrne, P. J. Hasnip, M. I. J. Probert

**Affiliations:** 1 School of Physics, Engineering and Technology, University of York, York, YO10 5DD UK; 2 Faculty of Sciences, Department of Physics, Sakarya University, 54050 Sakarya, Turkey; 3 Center for Advanced Laser Techniques, Institute of Physics, 10000 Zagreb, Croatia

**Keywords:** Density functional theory, Superconducting properties and materials

## Abstract

Using first principles calculations, we show that $\hbox {Li}_x\hbox {Al}_y\hbox {B}_{2(x+y)}$ materials have strong electron-phonon coupling, with many having a superconducting critical temperature ($T_c$) that exceeds that of the more familiar $\hbox {MgB}_2$ at ambient pressure. In particular, we find that $\hbox {LiAlB}_4$ is the most stable member of the family, with $T_c > 44\,\hbox {K}$ whilst the peak $T_c$ is with $\hbox {Li}_3\hbox {AlB}_8$ which has $T_c > 77\,\hbox {K}$. Our results reveal that these materials are both thermodynamically and dynamically stable, with strong electron-phonon coupling, indicating significant potential for practical superconducting applications.

## Introduction

The discovery of magnesium diboride ($\hbox {MgB}_2$) as a superconducting material in 2001^1,2^ exceeded expectations for the predicted limitations of critical temperatures ($T_c$) within the Bardeen-Cooper-Schrieffer (BCS) theory of superconductivity^3^. The structure of $\hbox {MgB}_2$ consists of monatomic layers of honeycomb-structured boron sheets and magnesium atoms, which are centred above each boron ring. It crystallizes in the P6/mmm space group (No. 191) with the layers perpendicular to the *c* lattice direction. The electronic states are analogous to benzene, where the $\sigma$-bonds are constrained by the boron planes to form covalent bonds through $sp^2$-hybridised boron orbitals. A key ingredient to high $T_c$ lies with the honeycomb-structured boron sheets, within which the $E_{2g}$ phonon mode relates to the in-plane vibration of light boron atoms. With the $\sigma$-band concentrated along the B-B axes, this particular phonon mode causes significant distortion in the $\sigma$-bond network, shifting the electronic states and contributing significantly to the electron-phonon coupling within $\hbox {MgB}_2$. To date, $\hbox {MgB}_2$ has the highest experimental $T_c$ at ambient pressure for a BCS-type superconductor at 39 K. Inspired by this, we consider materials that are isostructural to $\hbox {MgB}_2$ in search of similar electron-phonon coupling, whilst incorporating lighter elements in order to achieve higher critical temperatures.

In order to be able to rapidly evaluate many potential materials, we used first principles calculations based upon Density Functional Theory (DFT). In particular, we used the CASTEP code^4^ which has recently gained the ability to calculate the electron-phonon coupling parameter, and we validated this approach (see ’Validation Study’ section in the Supplementary Material for details) using the Quantum Espresso (QE) package^5,6^ which has a well-established electron-phonon coupling capability. Using CASTEP, we evaluated the binding enthalpy and structural stability of various candidate borides, and in particular, the ordered Li–Al–B system. We used the CASTEP Genetic Algorithm (GA)^7^ to find the global minimum energy structure of $\hbox {LiAlB}_4$, which was indeed found to be isostructural to $\hbox {MgB}_2$. We then evaluated the superconducting properties of this material using CASTEP (see below for details) and found $T_c = 49\,\hbox {K}$. As this was an extraordinarily high value, we validated the calculation by simulating the same structure with QE, and found $T_c = 44\,\hbox {K}$ - in remarkable agreement with CASTEP, given the exponential sensitivity of $T_c$ to the different parameters used with two independent codes. This validation of the results gave us confidence in the prediction that the $T_c$ of $\hbox {LiAlB}_4$ is significantly above that of $\hbox {MgB}_2$.

**Figure 1:**
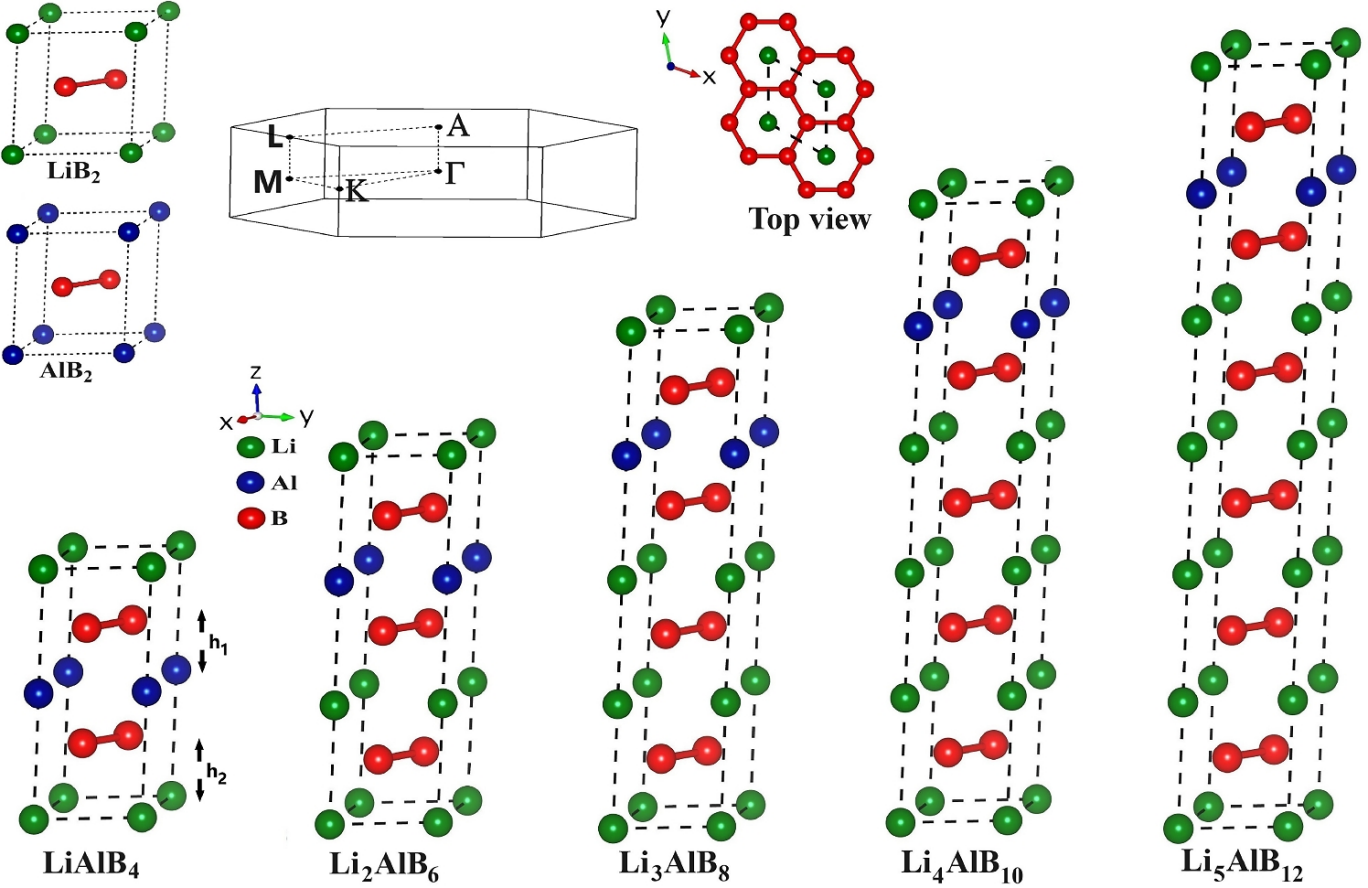
Schematic representation of the hexagonal crystal structure of $\hbox {Li}_x\hbox {Al}_y\hbox {B}_{2(x+y)}$. Li, Al, and B atoms are represented in green, blue, and red. The unit-cell is shown with dashed black lines. Additionally, top-view projections of these crystals and their corresponding Brillouin zones are provided.

Finally, we then used the CASTEP Convex Hull GA^8^ (CHGA) to search the full Li–Al–B ternary space in order to find other candidate structures (see Convex Hull GA Section in the Supplementary Material for details), and used QE to evaluate their $T_c$. A number of thermodynamically stable candidates of the general form $\hbox {Li}_x\hbox {Al}_y\hbox {B}_{2(x+y)}$ were found, with the same $\hbox {MgB}_2$ type structure, as shown in Fig. 1. Analysis of the binding enthalpy showed that $\hbox {LiAlB}_{4}$ was the most stable, with very little energy difference between the ‘ordered’ P6/mmm structures, comprising separate layers of Li and Al, and the ‘disordered’ P6/mmc structures with mixtures of Li and Al in each non-B layer. For $x\ge 2$ we found that the CHGA favoured $\hbox {Li}_x\hbox {Al}_y\hbox {B}_{2(x+y)}$ structures with mixed Li/Al layers ($\hbox {P6}_3$/mmc structures) which would then have additional entropic stabilization at finite temperatures as there are many more possibilities. In this paper we focus on the ‘ordered’ P6/mmm structures with monatomic Li and Al layers for convenience, and to keep the number of structures manageable. We have found that the family of ordered $\hbox {Li}_x\hbox {Al}_y\hbox {B}_{2(x+y)}$ materials has great superconductivity potential, and that $\hbox {LiAlB}_4$ is the most stable member of the family, with $T_c > 44\,\hbox {K}$ whilst $\hbox {Li}_3\hbox {AlB}_8$ has $T_c$ approaching 80 K. The significance of this is that the $T_c$ of $\hbox {LiAlB}_4$ exceeds that of $\hbox {MgB}_2$, whilst the $T_c$ of $\hbox {Li}_3\hbox {AlB}_8$ even exceeds the boiling point of liquid $\hbox {N}_2$ at 77 K. We find that $x\ge 2$ can have even higher $T_c$ values, although there may be experimental challenges in growing these phases.

## Results

### Structure

Inspired by $\hbox {MgAlB}_4$, a known superconductor with a $T_c$ of 12 K^9^, we considered layered materials with the structure M-B-Al-B, where M=Mg, Li, Ca, Na or Be, and each layer contains a single type of atom. We considered $\hbox {YCrB}_4$ type structures, as Tayran *et al.*^10^ has shown these to be thermodynamically stable, and also $\hbox {MgB}_2$ type structures. Only for $\hbox {MgAlB}_4$ and $\hbox {LiAlB}_4$ did we find that the $\hbox {MgB}_2$-type structure was more stable. Since $T_c$ is usually higher for lighter elements, we chose $\hbox {LiAlB}_4$ to investigate in more detail.

$\hbox {LiAlB}_4$ has a honeycomb boron atomic layer that is sandwiched between layers of Li and Al, which is structurally similar to $\hbox {MgB}_2$. The optimized structure of $\hbox {LiAlB}_4$ shows that it crystallizes in the hexagonal P6/mmm space group (No. 191) with Wyckoff positions for atoms at Li (1a) (0,0,0), Al (1b) (0,0,1/2) and B (4h) (1/3,2/3,$\hbox {z}_B$). Thus, the crystal structure of $\hbox {LiAlB}_4$ is determined by three different parameters: two crystal lattice parameters (*a* and *c*) and one internal parameter ($\hbox {z}_B$). Full structural parameters are given in Table S2 of the Supplementary Material.

**Figure 2:**
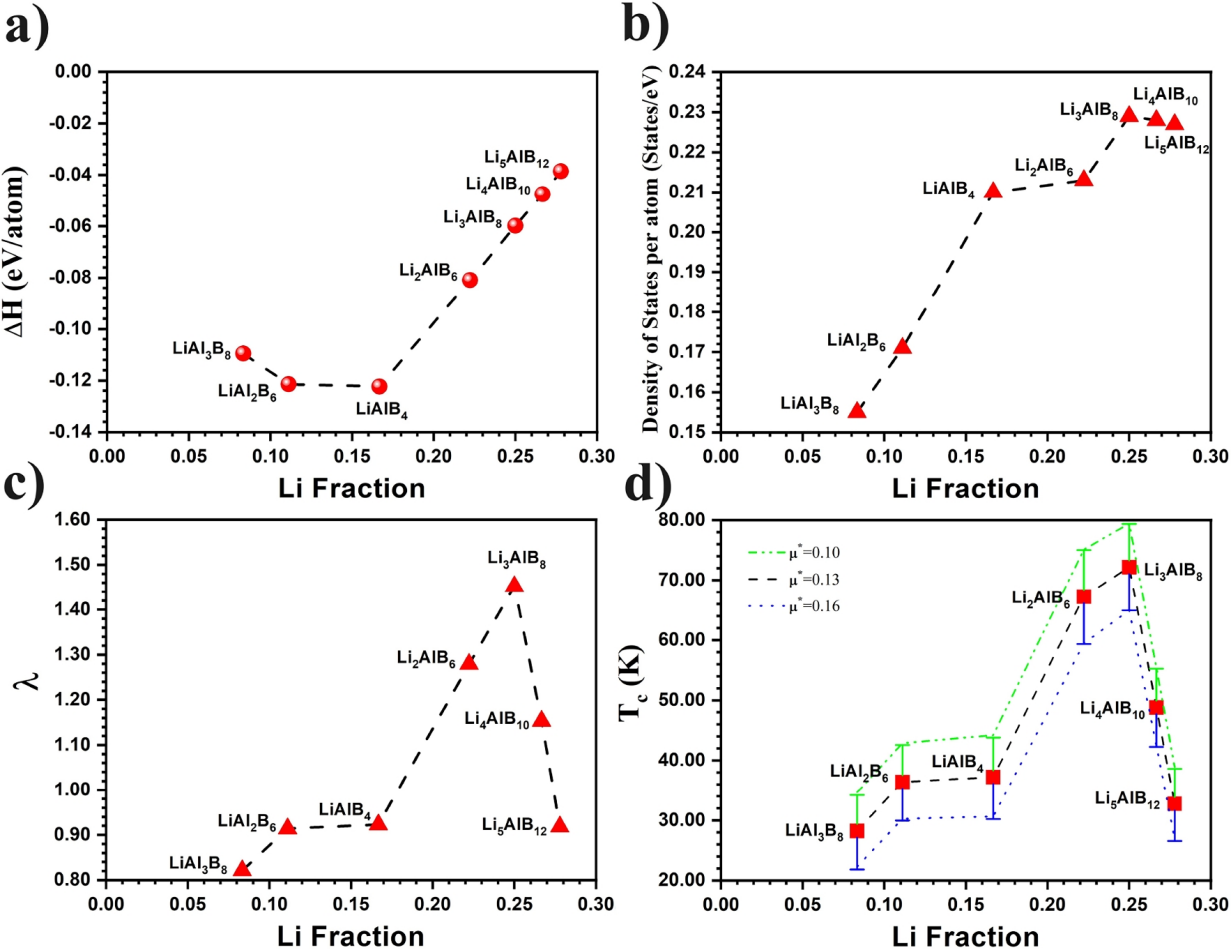
Li fraction dependence of (**a**) the binding enthalpy, (**b**) electronic density of states at the Fermi level, (**c**) average electron-phonon coupling constant ($\lambda$), (**d**) superconducting temperature ($T_c$) for the likely range of $\mu ^{*}$ values ($0.10 \dots 0.16$).

In order to compare different structures with different stoichiometries, we used the binding enthalpy per atom as in Ref.^11^. This is defined as:

1 $$\begin{aligned} \Delta H = \frac{1}{N}\left( E_\textrm{total}-\sum _{\alpha }{N_{\alpha }\mu _{\alpha }}\right) , \end{aligned}$$

where $E_\textrm{total}$ is the total calculated enthalpy of a particular cell of Li–Al–B system, *N* is the total number of atoms in the cell, $N_{\alpha }$ is the number of atoms of species $\alpha$ and $\mu _{\alpha }$ is the chemical potential of species $\alpha$. The binding enthalpy of a stable compound is negative, and the results are shown in Fig. 2. Details of the binding enthalpy, lattice parameters and bond lengths are given in Table S2 of the Supplementary Material. In general, the interlayer distance of Li-B is slightly larger than the interlayer distance in $\hbox {MgB}_2$, while the Al-B distance is quite small. The boron bonding is strongly anisotropic and similar to graphite.

We find that the $\hbox {LiAlB}_4$ has the lowest formation enthalpy of all the structures considered in this work, implying that it is more stable. Hence this material is the primary focus of our study. The other ordered phases (see below) are also thermodynamically stable, and might also be realized experimentally under appropriate growth conditions.

### Electronic structure

**Figure 3:**
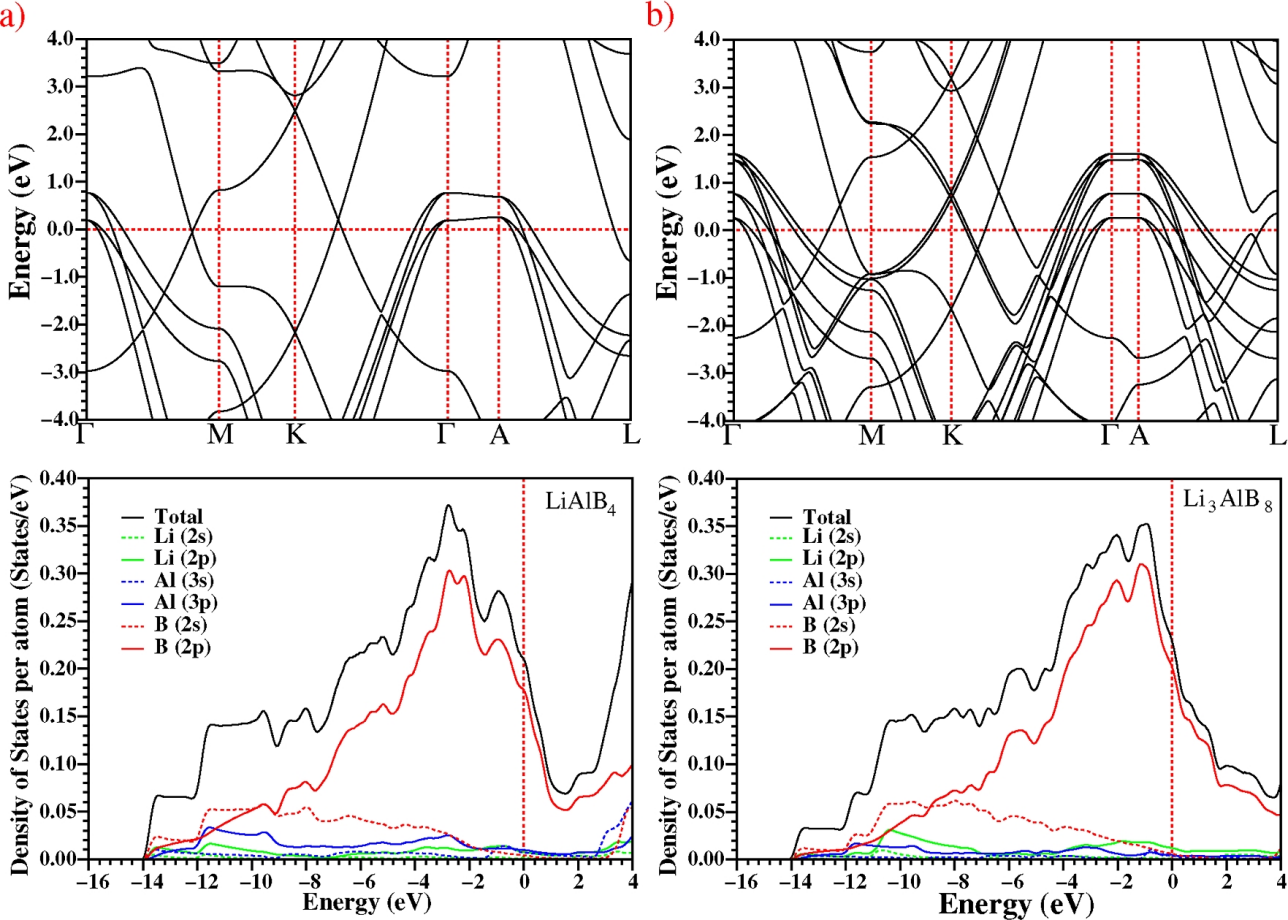
Upper panel: The calculated electronic band structure along the high symmetry directions in the first Brillouin zone; lower panel: calculated total and partial electronic density of states for (**a**) $\hbox {LiAlB}_4$ and (**b**) $\hbox {Li}_3\hbox {AlB}_8$.

The electronic properties of $\hbox {LiAlB}_4$ are given in Fig. 3, including the band structure in the Brillouin zone and the total and partial densities of states (DOS and PDOS). The electronic band structure and DOS of $\hbox {LiAlB}_4$ are similar to that of its isostructural counterpart $\hbox {MgAlB}_4$^11^. It is clear that $\hbox {LiAlB}_4$ is metallic, with five energy bands crossing the Fermi level at different points (“**k**-points”) in the Brillouin zone. The $\hbox {LiAlB}_4$ material has practically no dispersion in the $\Gamma -A$ direction, as expected due to the large separation between boron layers. The band structure of P6/mmm-$\hbox {LiAlB}_4$ exhibits a quasi-2D character, similar to that of $\hbox {MgB}_2$. This similarity suggests the possibility of excellent superconductivity, as $\hbox {MgB}_2$ has three energy bands crossing the Fermi level, whilst $\hbox {LiAlB}_4$ has five. In addition, $\hbox {MgB}_2$ has a doubly degenerate flat band near the Fermi level in the $\Gamma -A$ direction, while $\hbox {LiAlB}_4$ contains two doubly degenerate flat bands. Thus, the electronic density of states at the Fermi level ($N(E_F)$) of $\hbox {LiAlB}_4$ could be higher than $\hbox {MgB}_2$. Figure 3 shows the total and partial densities of states for $\hbox {LiAlB}_4$ to better understand the electronic structure. The PDOS is dominated by the orbital hybridization of B 2*p* below the Fermi level, indicating strong B-B covalent bonding. In addition, the Al *p* and B *s* orbitals below the Fermi level have similar behaviours, suggesting strong bonding between Al-B atoms. Corresponding data for $\hbox {MgB}_2$ is provided in Fig. S3. of the Supplementary Material.

The electronic density of states at the Fermi level ($N(E_F)$) is crucial for metallic phases and superconductivity, as Cooper pairs of electrons have energies close to the Fermi level. The value of the electron-phonon coupling parameter ($\lambda$) is directly proportional to $N(E_F)$, according to the McMillan-Hopfield expression^12^:

2 $$\begin{aligned} \lambda =\frac{N(E_{F})\langle I^2\rangle }{\langle M\rangle \langle \omega ^2\rangle } \end{aligned}$$

where $\langle \omega ^2\rangle$ denotes the average squared phonon frequency, $\langle I^2\rangle$ describes the average squared electron-phonon matrix element and $\langle M\rangle$ is the average atomic mass. The density of states (DOS) of $\hbox {LiAlB}_4$ is determined to be 1.261 states/eV per formula unit, which, as expected, is greater than that of $\hbox {MgB}_2$ (0.561 states/eV per formula unit). Since the mean mass of $\hbox {LiAlB}_4$ is also lower than that of $\hbox {MgB}_2$, the McMillan-Hopfield expression suggests that $\hbox {LiAlB}_4$ should have a larger electron-phonon coupling parameter (and hence $T_c$) than $\hbox {MgB}_2$. The density of states (DOS) in $\hbox {LiAlB}_4$ and $\hbox {MgB}_2$ materials is dominated by B 2*p* states, contributing approximately 85% (1.063 states/eV) and 91% (0.511 states/eV), respectively. It is clear that boron atoms play a crucial role in the phenomenon of superconductivity in these materials. The contribution of Li at the Fermi level is negligible.

**Figure 4:**
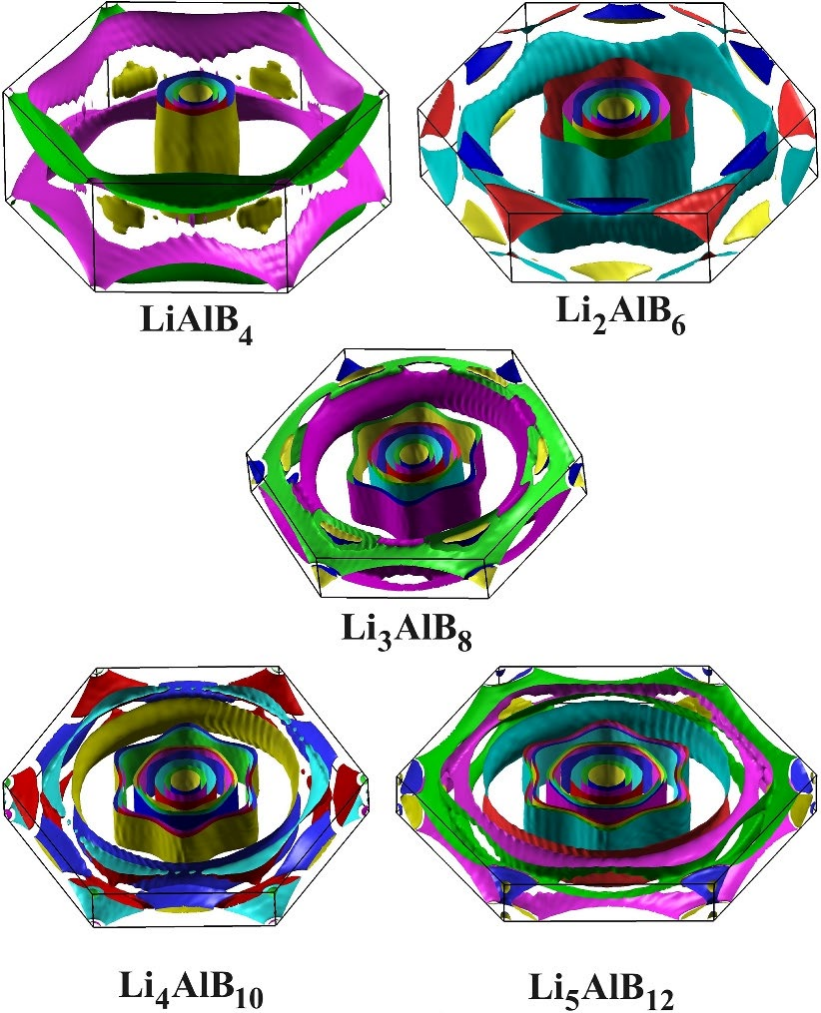
The Fermi surface of $\hbox {Li}_x\hbox {Al}_y\hbox {B}_{2(x+y)}$ for $1\le x \le 5$ and $y=1$ rendered using XCrySDen^35^ v1.6.2 from http://www.xcrysden.org.

The Fermi surface sheets and the Brillouin zone of $\hbox {LiAlB}_4$ are shown in Fig. 4. The four $\sigma$-bonding bands cross the Fermi level, as shown in Fig. 3, creating four cylindrical Fermi sheets surrounding the $\Gamma$ point and along the $\Gamma$-A direction. The Fermi surface of the $\pi$-band consists of planar honeycomb tubular networks. In quasi-two-dimensional (2D) systems, cylindrical Fermi sheets lead to a strong electron-phonon coupling with a specific phonon mode^13–15^. The existence of twice as many cylindrical Fermi surfaces in $\hbox {LiAlB}_4$, compared to $\hbox {MgB}_2$^16,17^, also suggests potentially strong electron-phonon coupling. This could lead to an anisotropic, multi-gap superconducting state, as found in $\hbox {MgB}_2$. Kafle *et al.* found in their study on Li-B compounds, isostructural to $\hbox {MgB}_2$, that the anisotropic Migdal-Eliashberg formalism increased the $T_c$ value by nearly three times compared to the isotropic calculations^18^. In this work, we have only used the isotropic approximation, and as such, even higher values of $T_c$ might be found with the anisotropic Migdal-Eliashberg formalism. This would be an interesting topic for further study.

### Phonons

**Figure 5:**
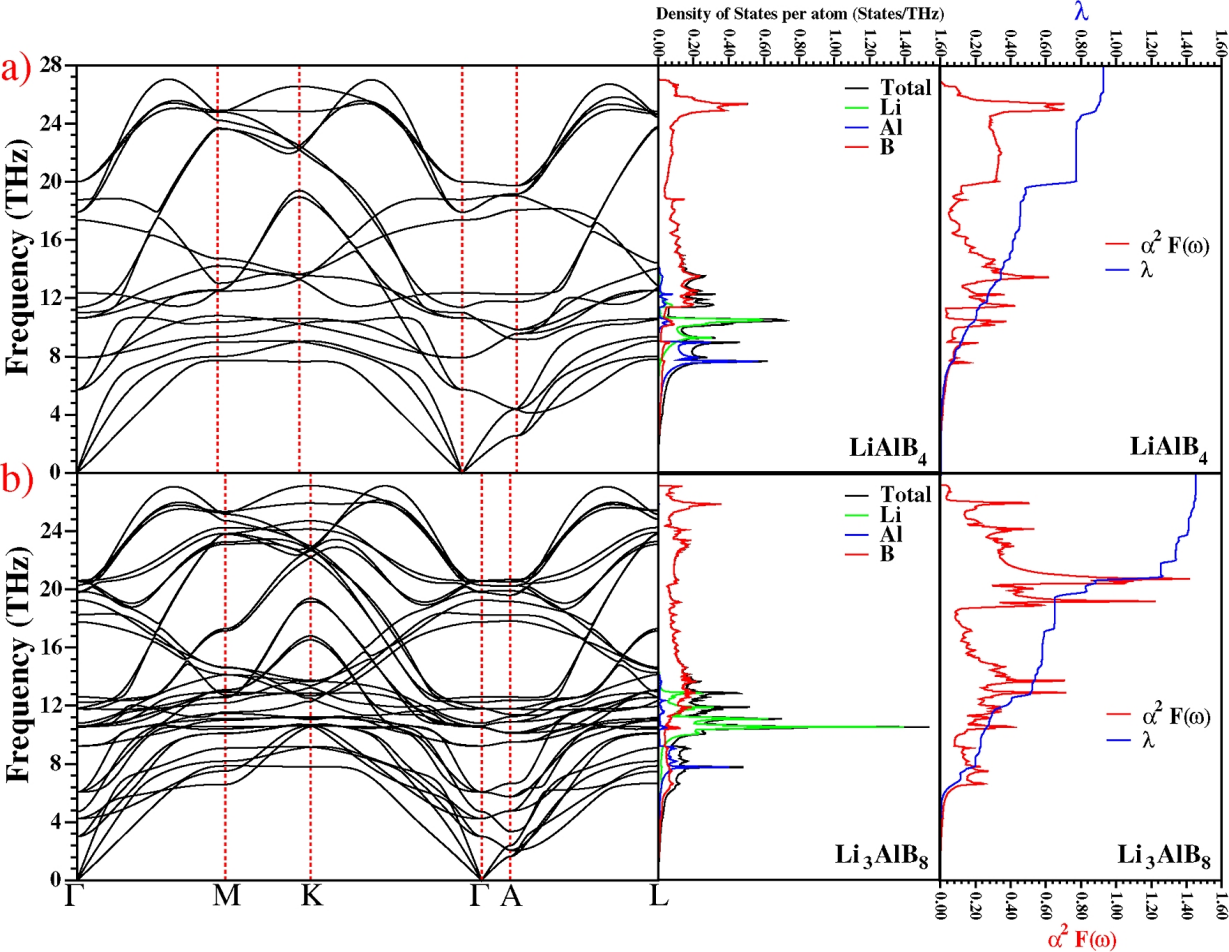
Phonon dispersion curves, total, partial vibrational density of states and the calculated electron-phonon spectral function $\alpha ^2 F(\omega )$ (red line) and the variation of the electron-phonon coupling parameter (blue line) with rising frequency $\lambda$($\omega$) for (**a**) $\hbox {LiAlB}_4$ and (**b**) $\hbox {Li}_3\hbox {AlB}_8$. Note that the different unit cell parameters of $\hbox {LiAlB}_4$ and $\hbox {Li}_3\hbox {AlB}_8$ mean that the spacing of $\Gamma$-M etc. is different, and hence the red dashed vertical lines that show the special points in the Brillouin zone do not exactly line up in the (**a**) and (**b**) panels.

The examination of phonon dispersion, total and partial phonon density of states (PhDOS), and electron-phonon spectral function of any superconductor is crucial because they play an important role in superconductivity. The $\hbox {LiAlB}_4$ compound has a primitive unit cell of six atoms and hence 18 phonon modes, consisting of three acoustic modes and 15 optical modes. The phonon spectrum and PhDOS for $\hbox {LiAlB}_4$ are shown in Fig. 5; the absence of imaginary frequencies indicates that $\hbox {LiAlB}_4$ is dynamically stable.

The vibrational modes can be divided into three sections: a low-frequency region up to 9.1 THz; a medium-frequency region ranging from 9.1 to 10.8 THz; and a high-frequency region above 10.8 THz. The low-frequency region, which contains the acoustic and low-frequency optic branches, mainly comprises vibrational modes of Al, with minor contributions from Li and B atoms, at a similar frequency to those found in $\hbox {MgB}_2$ for Mg-related modes. In the medium-frequency region, Li vibrations are dominant, with a minor contribution from Al-B hybridization. The high-frequency region is formed by the vibrations of B-B atoms. In the 20-27 THz region, two phonon branches ($\hbox {B}_{1g}$, $\hbox {E}_{2g}$) originating from B atoms show phonon anomalies in the $\Gamma$-M, K-$\Gamma$, and $\Gamma$-A directions.

The electron-phonon coupling parameters for the $\hbox {E}_{2g}$ phonon mode are significantly larger than those for the other phonon modes, which is similar to $\hbox {MgB}_2$. In addition, for $\hbox {LiAlB}_4$, the electron-phonon coupling parameter of the $\hbox {A}_{1g}$ and $\hbox {B}_{1g}$ modes, which originate from the B atoms, has a significantly large value at the $\Gamma$ point. The electron-phonon coupling parameters of IR-active optical phonon modes at the $\Gamma$ point are zero. A detailed analysis of the optical phonon modes at the zone centre is given in the ’Additional Phonon Analysis of $\hbox {LiAlB}_4$’ section of the Supplementary Material.

Our phonon dispersion for $\hbox {LiAlB}_4$ closely resembles that of $\hbox {MgB}_2$; however, $\hbox {MgB}_2$ exhibits anomalies just in the doubly degenerate $\hbox {E}_{2g}$ mode^19–22^. The presence of more phonon anomalies in $\hbox {LiAlB}_4$ (and multiple flat bands in the electronic band structure along the $\Gamma$-A direction) suggests that it may have a larger electron-phonon coupling, and thus a considerably higher $T_c$.

### Superconducting properties

Following from the discussion of vibrational properties, Fig. 5 shows the Eliashberg spectral function ($\alpha ^2F(\omega )$) and the frequency variation of the average electron-phonon coupling parameter ($\lambda$) for $\hbox {LiAlB}_4$. We find $\lambda$ for $\hbox {LiAlB}_4$ to be 1.104 and 0.923 with CASTEP and QE (see Table 1), respectively. The difference in $\lambda$ obtained from the two programs may be due to the different pseudopotentials used and k-point settings (see Method section for details). The phonon modes in the low-frequency region are dominated by Al atoms, contributing 12% to the total $\lambda$. The Migdal-Eliashberg theory predicts that low-frequency phonon modes will significantly contribute to the electron-phonon interaction (see Eq.4); however, this is not the case for the $\hbox {LiAlB}_4$ material, suggesting that Al atoms do not play an important role in the superconducting mechanism. This is consistent with the findings of Li *et al.* for $\hbox {Mg}_{1-x}\hbox {Al}_x\hbox {B}_2$, where $T_c$ gradually decreased with increasing Al concentration^9^. The phonon density of states exhibits a strong peak related to Li atoms in the narrow frequency range of 9.1 THz to 10.8 THz. This region contributes 10% to the total $\lambda$. This medium-frequency contribution is lacking in $\hbox {MgB}_2$, for which the Mg-related modes have low coupling. The results suggest that a higher Li ratio will lead to increased $\lambda$ and, thus, $T_c$. The contribution of high-frequency phonon vibrations above 10.8 THz, mostly related to the B-B atoms, is 78%, implying that superconductivity in $\hbox {LiAlB}_4$ is mainly controlled by boron modes, as is also the case with $\hbox {MgB}_2$. We can therefore conclude that the origin of superconductivity is similar in each of these compounds. The logarithmic average phonon frequencies calculated with CASTEP and QE are 614.4 and 722.3 K, respectively. As discussed (see ’Validation Study’ section in the Supplementary Material), we found the best fit $T_c$ for $\hbox {MgB}_2$ was with $\mu ^{*} = 0.10$ according to the Allen-Dynes modification of the McMillan formula. Using the same value of $\mu ^{*}$ for $\hbox {LiAlB}_4$, we predict $T_c = 44.21\,\hbox {K}$ with QE and $49.39\,\hbox {K}$ with CASTEP. In general, reasonable values for $\mu ^{*}$ are in the range 0.10 to 0.16. Using $\mu ^{*} = 0.16$ we find $T_c$ is 30.63 K for $\hbox {LiAlB}_4$, which is therefore the lower-limit of theoretically likely values. In addition, we observe that for the other materials studied, $T_c$ decreases as $\mu ^{*}$ increases (see Table S3 of the Supplementary Material) as shown in Fig. 2d.

**Table 1:** The logarithmic frequency ($\omega _{\ln }$), the average electron-phonon coupling parameter ($\lambda$) and the superconducting transition temperature ($T_c$ in K) using $\mu ^{*} = 0.10$ for the hexagonal Li–Al–B system and compared with previous theoretical results.

Material	$\omega _{\ln }$ (K)	$\lambda$	$T_{c}$ $\hbox {(K)}^a$	Eliashberg $T_{c}$ $\hbox {(K)}^b$
${\textbf {AlB}}_2$	**476.2**	**0.529**	**7.18**	4.923
Ref.^27^ (GGA with QE)		0.430	3.43	
${\textbf {LiAl}}_3{\textbf {B}}_8$ (GGA with QE)	**702.2**	**0.821**	**34.66**	38.958
${\textbf {LiAl}}_2{\textbf {B}}_6$ (GGA with QE)	**647.3**	**0.914**	**42.87**	49.359
${\textbf {LiAlB}}_4$ (GGA with CASTEP)	**614.4**	**1.104**	**49.39**	
${\textbf {LiAlB}}_4$ (GGA with QE)	**722.3**	**0.923**	**44.21**	57.277
${\textbf {Li}}_2{\textbf {AlB}}_6$ (GGA with QE)	**778.2**	**1.279**	**75.14**	80.604
${\textbf {Li}}_3{\textbf {AlB}}_8$ (GGA with QE)	**718.9**	**1.452**	**79.42**	88.167
${\textbf {Li}}_4{\textbf {AlB}}_{10}$ (GGA with QE)	**650.3**	**1.153**	**55.38**	57.839
${\textbf {Li}}_5{\textbf {AlB}}_{12}$ (GGA with QE)	**642.4**	**0.918**	**38.93**	46.266
${\textbf {LiB}}_2$ (GGA with QE)	**641.1**	**0.848**	**33.71**	28.492
Ref.^26^ (GGA with QE)	621.0	0.830	31.40	

^a^Value calculated using standard Allen-Dynes equation.

^b^ Value calculated using a2Tc with full Eliashberg method

### Superstructures

Having demonstrated the superconducting potential of $\hbox {LiAlB}_4$, we used the CASTEP genetic algorithm^7^ and its recently developed convex hull functionality^8^ to search the Li–Al–B ternary structure space for other stable structures, with a particular focus on increasing proportions of the lighter Li atoms. A number of thermodynamically stable candidates of the general form $\hbox {Li}_x\hbox {Al}_y\hbox {B}_{2(x+y)}$ were found, with the same $\hbox {MgB}_2$ type structure (see Convex Hull GA Section in the Supplementary Material for details). For $\hbox {LiAlB}_{4}$ we found that there was very little energy difference between the ‘ordered’ P6/mmm structures, with separate layers of Li and Al, and the ‘disordered’ P6/mmc structures with mixtures of Li and Al in each non-B layer. In this paper, we focus on the ‘ordered’ P6/mmm structures with monatomic Li and Al layers for convenience and to keep the number of structures manageable; however, we note that for $x\ge 2$ the CHGA favoured $\hbox {Li}_x\hbox {Al}_y\hbox {B}_{2(x+y)}$ structures with mixed Li/Al layers ($\hbox {P6}_3$/mmc structures), which have higher configurational entropy and so could be stabilised further at finite temperatures. Figure 2a shows the variation in binding enthalpy for different possible superstructures in the ordered $\hbox {Li}_x\hbox {Al}_y\hbox {B}_{2(x+y)}$ family, as the Li fraction changes. The lowest formation enthalpy of all was for the $\hbox {LiAlB}_4$ structure, which suggests that this compound is more stable compared to other Li–Al–B systems, although other ordered phases are possible.

The electronic band structure and Fermi surfaces of these potential superconductors have been investigated in detail, and found to be broadly similar across the $\hbox {Li}_x\hbox {Al}_y\hbox {B}_{2(x+y)}$ ordered family of systems. For each value of *x*, there are two more bands that cross the Fermi level, and an extra doubly degenerate flat band appears in the $\Gamma -A$ direction. Figure 2b shows the change in N($\hbox {E}_F$) per atom as a function of the Li fraction. The highest value of N($\hbox {E}_F$) per atom is found in $\hbox {Li}_3\hbox {AlB}_8$, which is nearly identical to $\hbox {Li}_4\hbox {AlB}_{10}$ and $\hbox {Li}_5\hbox {AlB}_{12}$. The Fermi surfaces of these materials are shown in Fig. 4. The circular Fermi sheet originating from $\hbox {Li}_4\hbox {AlB}_{10}$ and $\hbox {Li}_5\hbox {AlB}_{12}$ is further away from the $\Gamma -A$ direction, indicating a decrease in the electron-phonon interaction along the $\Gamma -A$ direction compared to $\hbox {Li}_3\hbox {AlB}_8$. Additionally, the Fermi nesting of $\hbox {Li}_3\hbox {AlB}_8$ is stronger than the other materials, particularly along the K-M-L direction. The significant Fermi nesting seen in $\hbox {Li}_3\hbox {AlB}_8$ reveals stronger electron-phonon interactions, potentially resulting in the highest $T_c$^23–25^.

The phonon properties of all $\hbox {Li}_x\hbox {Al}_y\hbox {B}_{2(x+y)}$, like their electrical properties, show broadly similar characteristics. In Fig. 5a and b, the phonon properties of $\hbox {LiAlB}_4$ (the most thermodynamically stable) and $\hbox {Li}_3\hbox {AlB}_8$ (the greatest electron-phonon coupling) are presented. For $\hbox {Li}_3\hbox {AlB}_8$, two additional phonon branches in the 20-25 THz range, originating from boron atoms, exhibit phonon anomalies, indicating stronger electron-phonon interactions. As seen in Fig. 5, the electron-phonon coupling parameter of $\hbox {Li}_3\hbox {AlB}_8$ exhibits a sudden increase from 0.88 to 1.26, contributing 26% to the total $\lambda$. All phonon branches for $\hbox {Li}_3\hbox {AlB}_8$ are approximately 0.2 THz lower compared to those of $\hbox {Li}_4\hbox {AlB}_{10}$ and $\hbox {Li}_5\hbox {AlB}_{12}$. It is these lower phonon frequencies, combined with a high $N(E_F)$ and strong Fermi nesting, which leads to its strong electron-phonon interactions. In Table 1, we present the $\lambda$ and $T_c$ values for all materials. As expected, $\hbox {Li}_3\hbox {AlB}_8$ has the highest $T_c$, calculated to be $79.42\,\hbox {K}$, assuming $\mu ^{*} = 0.10$ as before. This value is remarkably higher than that of $\hbox {MgB}_2$ and notably exceeds the boiling point of liquid nitrogen (77.3 K), highlighting its potential for practical superconducting applications. Whilst this value of $\mu ^{*}$ gave the best fit for the $T_c$ of $\hbox {MgB}_2$, $\mu ^{*}$ is material-dependent, with the common range being from 0.10 to 0.16. We find that increasing $\mu ^{*}$ reduces $T_c$. However, even the largest $\mu ^{*}= 0.16$ gives $T_c = 64.98\,\hbox {K}$ for $\hbox {Li}_3\hbox {AlB}_8$, which is still significantly higher than that of $\hbox {MgB}_2$. For completeness, we show the effect of different values of $\mu ^{*}$ on $T_c$ in Fig. 2, for all the candidate $\hbox {Li}_x\hbox {Al}_y\hbox {B}_{2(x+y)}$ structures. We also considered the two natural end-point structures, $\hbox {LiB}_2$ and $\hbox {AlB}_2$, both having P6/mmm symmetry. We find that $\hbox {LiB}_2$ ($T_c=33.71\,\hbox {K}$) has a much higher $T_c$ than $\hbox {AlB}_2$ ($T_c=7.18\,\hbox {K}$), which is in excellent agreement with previous theoretical values^26,27^. All the calculated $T_c$ values are given in Table 1 .

For all the $\hbox {Li}_x\hbox {Al}_y\hbox {B}_{2(x+y)}$ materials we find $\lambda \ge 0.8$ which corresponds to strong coupling. The usual Allen-Dynes approach to $T_c$ is a weak coupling approximation, and hence we also calculate $T_c$ for all these materials using the full Eliashberg method with C.J. Pickard’s ‘a2Tc’ code. These results are also included in Table 1 . For the binary materials, using a2Tc reduces the predicted $T_c$, and for the ternaries it increases it. This confirms our overall conclusion of unusually high $T_c$ in the Li–Al–B system.

## Summary

We have investigated the physical and superconducting properties of stable ordered P6/mmm structures in the Li–Al–B system using first-principles methods. We find that the $\hbox {Li}_x\hbox {Al}_y\hbox {B}_{2(x+y)}$ materials have a lot of promise as ambient pressure superconductors due to their unusually strong electron-phonon coupling. The electron-phonon interaction is studied for the first time using the CASTEP code, and it was found to be consistent with results obtained from Quantum Espresso. Our results indicate that $\hbox {Li}_x\hbox {Al}_y\hbox {B}_{2(x+y)}$ compounds are thermodynamically and dynamically stable. Notably, our results show that $\hbox {LiAlB}_4$ is the most stable and has $T_c = 44.21\,\hbox {K}$, whilst $\hbox {Li}_3\hbox {AlB}_8$ has the highest superconducting critical temperature with $T_c = 79.42\,\hbox {K}$ with $\mu ^{*} = 0.10$, surpassing even liquid nitrogen temperatures. This promising result underscores the need for experimental validation of our results.

## Method

The binding enthalpy for each different structure was evaluated in *ab initio* calculations using density functional theory (DFT) in the CASTEP code^4^ using the PBE approximation^28^ and norm-conserving pseudopotentials^29^. Following convergence tests, the CASTEP PBE calculations used a $27 \times 27 \times 12$ **k**-point Monkorst-Pack (MP) grid with a plane-wave cut-off energy of 900 eV and a grid size of 2$G_{max}$ [except for the high-throughput CASTEP-GA calculations, which used a slightly lower plane-wave cut-off energy of 700 eV for speed]. The electronic properties were calculated with a fine **k**-point MP grid of $54 \times 54 \times 24$, while phonon properties were calculated using Density Functional Perturbation Theory^30^ on a $9 \times 9 \times 4$ **q**-point MP grid with $27 \times 27 \times 12$ **k**-points. The phonon interpolation was carried out across 10,000 Fermi surface points and the electron-phonon coupling calculation on 243 *q*-vectors linking Fermi surface points.

With the CASTEP code, we find $T_c = 49.39\,\hbox {K}$ for $\hbox {LiAlB}_4$. A confirmatory calculation of the same structure with the Quantum Espresso (QE) code utilizing finer parameters (60 Ry cutoff for wave functions and 600 Ry for charge density, a $32\times 32 \times 16$ MP grid for the ground state and $40\times 40 \times 20$ MP grid for electronic and Fermi surface calculations) produced $T_c = 44.21\,\hbox {K}$.

To calculate the superconducting properties, we use the Migdal-Eliashberg approach^31,32^, as outlined in other works, e.g. Ref.^25^. This formalism has recently been implemented^33^ in CASTEP and was used to calculate the electron-phonon interaction and hence the critical temperature ($T_c$). In this formalism, the Eliashberg spectral function^12^ is described as follows:

3 $$\begin{aligned} \alpha ^2 F(\omega )=\frac{1}{2\pi N(E_{F})} \sum _{\textbf{q}j}\frac{\gamma _{\textbf{q}j}}{\hbar \omega _{\textbf{q}j}}\delta \left( \omega -\omega _{\textbf{q}j}\right) , \end{aligned}$$

where $N(E_F)$ is the electron density of states per atom and spin at the Fermi level, and $\alpha$ is the average of all phonons with energy $\omega$ in the BZ. The average electron-phonon coupling constant is determined by^12,34^:

4 $$\begin{aligned} \lambda (\omega )=2\int _{0}^{\infty } \frac{\alpha ^{2}F(\omega )}{\omega }d\omega . \end{aligned}$$

The value of $\lambda (\omega )$ is then used to determine the logarithmic average phonon frequency ($\omega _{\ln }$ ) using

5 $$\begin{aligned} \omega _{ln} = \exp \left( 2\lambda ^{-1}\int _{0}^{\infty } \frac{d\omega }{\omega }\alpha ^2F(\omega )ln\omega \right) . \end{aligned}$$

Finally, the Allen-Dynes modification of the McMillan formula is used with these values of $\lambda$ and $\omega _{\ln }$ to determine the superconducting transition temperature $T_c$:

6 $$\begin{aligned} T_{c}=\frac{\omega _\textrm{ln}}{1.2}\textrm{exp} \left( -\frac{1.04(1+\lambda )}{\lambda -\mu ^{*}(1+0.62\lambda )}\right) , \end{aligned}$$

where $\mu ^{*}$ represents an effective screened Coulomb repulsion parameter. The value of $\mu ^{*}$ typically ranges from 0.10 to 0.16 in most studies^12,34^. We use $\mu ^{*} = 0.10$ for all materials in this study, as this gave the best fit to experimental values of $T_c$ for $\hbox {MgB}_2$ - see ’Validation Study’ section and FIG. S1 in the Supplementary Material for details.

## Supplementary Information

Supplementary Information.

## Data Availability

The data created and analysed during the current study are available from https://doi.org/10.15124/f7a8d4f7-2f7b-4992-a84d-3dbeec36b995
