# Supplementary material for: Superconductivity in ordered Li–Al–B compounds
Source: Sci Rep. 2025 Jan 2;15:19. doi: 10.1038/s41598-024-84542-6 (PMC11696708; doi:10.1038/s41598-024-84542-6)
Supplement: Supplementary file 1 — Supplementary Information. [file 41598_2024_84542_MOESM1_ESM.pdf]

Supplementary Material: Superconductivity in Li-Al-B system

K. Hussain[1], S. J. Donaldson[1], E. Karaca[1,2,3], P. J. P. Byrne[1], P. J. Hasnip[1] and M. I. J. Probert[1]

[1] *School of Physics, Engineering & Technology, University of York, York YO10 5DD, United Kingdom*

[2] *Sakarya University, Faculty of Sciences, Department of Physics, 54050, Sakarya, Turkey*
and
[3] *Center for Advanced Laser Techniques, Institute of Physics, 10000 Zagreb, Croatia*

I. VALIDATION STUDY

In order to validate the electron-phonon coupling and T_c calculation in CASTEP, we validated the code on MgB_2. CASTEP v19 norm-conserving pseudopotentials were used with the Perdew-Burke-Ernzerhof (PBE) exchange-correlation functional[1]. Converged calculations used a Monkhorst-Pack (MP) grid of $24 \times 24 \times 12$ **k**-points and a cut-off energy of 1200 eV and a grid size of $2G_{max}$. The initial unit cell was based on experimental data[2] and then the LBFGS method was used to find the relaxed structure.

A finer sampling grid of $72 \times 72 \times 36$ was used in a density of states (DOS) calculation to find the electronic density of states at the Fermi level ($N(E_F)$) of 0.7041 states/eV. A density functional perturbation theory (DFPT) calculation was carried out for a **q**-point grid of $12 \times 12 \times 6$ with an offset of $\frac{1}{24}, \frac{1}{24}, \frac{1}{12}$. The generated phonon modes were interpolated across 10,000 Fermi surface points. An electron-phonon coupling calculation was performed on 200 **q**-vectors linking points on the Fermi surface. Through the Migdal-Eliashberg formalism for the Eliashberg spectral function, the T_c was calculated with the Allen-Dynes equation to be 39.16 K, showing excellent agreement with experimental findings, with ($\lambda = 1.011$) and $\mu^* = 0.10$. The value of μ^* for most BCS-type superconductors is in the range $0.10 \ldots 0.16$ and needs to be constrained by some other data. The full variation of T_c with μ^* can be seen in Fig. 1. On this occasion, we compare against the known experimental value of T_c for MgB_2 and hence choose $\mu^* = 0.10$ for this study. These results confirm that the electron-phonon coupling calculation and workflow with CASTEP is working as expected. Our equivalent calculation for MgB_2 using the Quantum Espresso package with $\mu^* = 0.10$ found $\lambda = 0.852$ and $T_c = 42.37$ K. This result is in excellent agreement with the T_c value obtained using the CASTEP code.

II. CONVEX HULL GENETIC ALGORITHM STUDY

In order to find candidate $Li_xAl_yB_{2(x+y)}$ structures and preliminarily assess the position of $LiAlB_4$ with respect to the convex hull, a ternary Li-Al-B convex hull was generated using the CASTEP convex-hull genetic algorithm (CHGA) [3]. As the number of geometry optimisation calculations required for a thorough search of the ternary potential energy surface is large, the pre-trained CHGNet Machine Learned (ML) potential [4] was utilised for all structural evaluations. This allowed multiple CHGA calculations with varying parameters to be carried out to ensure a reasonable exploration of the potential energy surface. This resulted in new candidate $Li_xAl_yB_{2(x+y)}$ structures being discovered that lie on (or near) the convex hull. These also contain the hexagonal boron sheets seen in MgB_2 and $LiAlB_4$. The results can be seen in Fig. 2, along an edge running between AlB_2 and LiB_2, and the $Li_xAl_yB_{2(x+y)}$

structures lie on this edge. $LiAlB_4$ is also shown, with a binding enthalpy very slightly above the CHGNet hull by 0.004 eV/ion, which is well within the range of thermal accessibility and ML accuracy.

The use of ML allows for relatively rapid structural searches. However, it is possible that any ML potential may not account for an unknown ground state structure and/or a novel structure may be predicted to be stable when it is not. As such, any ML results of interest must be validated with more robust *ab initio* methods; for this reason the convex hull with CHGNet was used only to propose new structures, which were then evaluated more rigorously with DFT (including the dynamic stability tests). See Method section in the main text for DFT details.

III. ADDITIONAL PHONON ANALYSIS OF LIALB$_4$

The primitive unit cell of $LiAlB_4$ contains 6 atoms, resulting in 18 phonon branches for any given wave vector, including three acoustic and 15 optical branches. The optical phonon modes at the zone center can be classified according to the irreducible representations of the D_{6h}(6/mmm) point group. Based on group theory, the symmetries of the optical zone-center phonon modes are:

$$\Gamma = A_{1g} + 2A_{2u} + B_{1g} + B_{2u} + E_{1g} + E_{2g} + 2E_{1u} + E_{2u}. \tag{1}$$

The A_{1g}, E_{1g}, and E_{2g} modes are Raman active (R), while the A_{2u} and E_{1u} modes are infrared active (IR). The remaining optical modes are silent. The one-dimensional A and B modes involve displacements along the **z** direction, and the doubly degenerate E modes involve displacements in the $\mathbf{x}-\mathbf{y}$ plane. Table III provides a comparative analysis of the zone-center phonon frequencies and electron-phonon coupling parameters for $LiAlB_4$. This shows that the electron-phonon coupling parameter of the E_{2g} phonon mode is considerably larger than that of the remaining phonon modes, closely resembling the behavior observed in MgB_2. The lowest frequency mode E_{1g}, as well as the A_{1g} and B_{1g} optical phonon modes originating from the B atoms, also make significant contributions. This is further evidence that the superconductivity of $LiAlB_4$ originates from the B atoms, indicating that the origin of superconductivity is similar to that in MgB_2, which have similar crystal structure. Phonons play a crucial role in the BCS superconductivity of these materials.

IV. ADDITIONAL STRUCTURAL, ELECTRONIC, PHONON AND ELECTRON-PHONON PROPERTIES OF LI$_x$AL$_y$B$_{2(x+y)}$ PHASES

We investigated the structural, electrical, phonon, and electron-phonon properties of $Li_xAl_yB_{2(x+y)}$. In addition, we examined the electronic properties of MgB_2 to compare with these new phases. The hexagonal structure of $Li_xAl_yB_{2(x+y)}$, which crystallizes in the P6/mmm space group, has been thoroughly examined. The main text discusses the detailed properties of $LiAlB_4$ and Li_3AlB_8. Table II shows the crystal lattice parameters and the lengths of the B-B bond lengths for all the materials considered in this study. The a and c lattice parameters for MgB_2, AlB_2 and LiB_2 consistent with previous theoretical and experimental values[5–8]. This consistency suggests that these calculations are also suitable for the novel $Li_xAl_yB_{2(x+y)}$ materials in the P6/mmm structure. Increasing the value of x slightly decreases the B-B distance, which could affect the bonding structure and superconductivity.

The thermodynamic stability of $LiAlB_4$ corresponds with its orthorhombic phase[9] and its isostructural compound, $MgAlB_4$[5]. The calculated crystal parameter a and c in Table II, performed using both CASTEP and QE, exhibit excellent agreement. Table II also contains data for the bond lengths of d_{B-B}. The Li-B bond-length is 2.483 Å, while the Al-B bond-length is 2.341 Å, indicating stronger Al-B bonding, given that Li has a much smaller ionic radius than Al. The number of layers per unit cell in $LiAlB_4$ is double that of MgB_2. The perpendicular distances

between the Li-B and Al-B in $LiAlB_4$ are h_2=1.778 and h_1=1.575 Å, respectively. The interlayer distance of Li-B is slightly larger than that of MgB_2, which is 1.76 Å[10], while the Al-B distance is quite small. The bond length between boron planes (3.149 Å) is significantly larger than the in-plane B-B (1.732 Å) distance, indicating a strong anisotropy similar to that seen in graphite.

Additional data on the properties of MgB_2, with the electronic properties are shown in Fig. 3, and the phonon dispersion properties in Fig. 4. MgB_2 exhibits phonon anomalies just in the doubly degenerate E_{2g} mode. Our results are extremely similar to previous theoretical work [11–14]. As mentioned in the main text, MgB_2 has only a single flat band around the Fermi level in the Γ-A direction and only one phonon mode exhibiting an anomaly.

The band structure, total and projected electronic local density of states of $LiAlB_4$ and Li_3AlB_8 are discussed in detail in the main text. $LiAlB_4$ has two flat bands and two phonon modes that exhibit anomalies. This suggests a stronger electron-phonon interaction, resulting in higher T_c. Here, we present the corresponding electronic structure data for $Li_xAl_yB_{2(x+y)}$ ($x = 2, 4$ and 5), as shown in Figs. 5, 6 and 7. These figures clearly show that all of these materials show metallic properties, with the B 2p states being the main contributors to the density of states at the Fermi energy $N(E_F)$. Thus, according to the McMillan-Hopfield formula, we can assume that the superconducting properties in these materials are mainly due to the boron atoms.

In Li_2AlB_6, in addition to the two double degenerate bands found in $LiAlB_4$, an extra double degenerate flat band appears about 1.5 eV (see Fig. 5). Similarly, extra doubly degenerate flat bands for Li_3AlB_8, Li_4AlB_{10}, and Li_5AlB_{12} have been found at about 1.5 eV.

Finally, the calculated phonon dispersion relations for $Li_xAl_yB_{2(x+y)}$ ($x = 2, 4$ and 5) are shown in Figs. 8, 9 and 10. These show that each of the materials are dynamically stable. In comparison to $LiAlB_4$, these materials have more flat bands near the Fermi level and more phonon mode anomalies, as discussed in detail in the main text.

REFERENCES

[1] J. P. Perdew, K. Burke, and M. Ernzerhof, Generalized gradient approximation made simple, Physical Review Letters **77**, 3865 (1996).

[2] S. Lee, H. Mori, T. Masui, Y. Eltsev, A. Yamamoto, and S. Tajima, Growth, structure analysis and anisotropic superconducting properties of MgB_2 single crystals, Journal of the Physical Society of Japan **70**, 2255 (2001).

[3] S. Donaldson, R. A. Lawrence, and M. I. J. Probert, A Genetic Algorithm For Convex Hull Optimisation , arXiv:2404.14354 [cond-mat.mtrl-sci] (2024).

[4] B. Deng, P. Zhong, K. Jun, J. Riebesell, K. Han, C. J. Bartel, and G. Ceder, CHGNet as a pretrained universal neural network potential for charge-informed atomistic modelling , Nature Machine Intelligence **5**, 1031-1041 (2023).

[5] F. Wang, J. Li, C. Shi, E. Liu, C. He, and N. Zhao, Comparison of electronic structures and mechanical properties of $MgAlB_4$, AlB_2 and MgB_2 using first-principles calculations, Ceramics International **46**, 12548 (2020).

[6] Y. Jiang and Y. Liang, Enhancing mechanical properties of MgB_2 superconductors through Nb substitution: A first-principles study, Physica B: Condensed Matter **683**, 415931 (2024).

[7] J. A. Alarco, P. C. Talbot, and I. D. Mackinnon, Coherent phonon decay and the boron isotope effect for MgB_2, Physical Chemistry Chemical Physics **16**, 25386 (2014).

[8] L. Hao, F. Ling, Y. Gu, P. Li, Y. Zhao, Y. Zhang, and D. Yu, First-principles study on novel LiB2 phases and superconductivity at ambient pressure, Chemical Physics **576**, 112093 (2024).

[9] C. Tayran, S. Aydin, M. Çakmak, and Ş. Ellialtıoğlu, Alkali and Alkaline earth metal doped aluminum tetraborides containing intrinsic planar boron sheet: $XAlB_4$ (X= Li, Mg, Ca, and Na), Computational Materials Science **124**, 130 (2016).

[10] J. Pešić, I. Popov, A. Šolajić, V. Damljanović, K. Hingerl, M. Belić, and R. Gajić, Ab initio study of the electronic,

vibrational, and mechanical properties of the magnesium diboride monolayer, Condensed Matter **4**, 37 (2019).

[11] Y. Kong, O. Dolgov, O. Jepsen, and O. Andersen, Electron-phonon interaction in the normal and superconducting states of MgB_2, Physical Review B **64**, 020501 (2001).

[12] A. Shukla, M. Calandra, M. d'Astuto, M. Lazzeri, F. Mauri, C. Bellin, M. Krisch, J. Karpinski, S. Kazakov, J. Jun, *et al.*, Phonon Dispersion and Lifetimes in MgB_2, Physical review letters **90**, 095506 (2003).

[13] A. Eiguren and C. Ambrosch-Draxl, Wannier interpolation scheme for phonon-induced potentials: Application to bulk MgB_2, W, and the (1× 1) H-covered W (110) surface, Physical Review B **78**, 045124 (2008).

[14] D. Novko, F. Caruso, C. Draxl, and E. Cappelluti, Ultrafast hot phonon dynamics in MgB_2 driven by anisotropic electron-phonon coupling, Physical review letters **124**, 077001 (2020).

[15] I. R. Shein and A. L. Ivanovskii, Elastic properties of mono-and polycrystalline hexagonal AlB_2-like diborides of s, p and d metals from first-principles calculations, Journal of Physics: Condensed Matter **20**, 415218 (2008).

[16] Y. Duan, Y. Sun, Z. Guo, M. Peng, P. Zhu, and J. He, Elastic constants of AlB_2-type compounds from first-principles calculations, Computational materials science **51**, 112 (2012).

[17] M. d'Astuto, R. Heid, B. Renker, F. Weber, H. Schober, O. De la Peña-Seaman, J. Karpinski, N. D. Zhigadlo, A. Bossak, and M. Krisch, Nonadiabatic effects in the phonon dispersion of $Mg_{1-x}Al_xB_2$, Physical Review B **93**, 180508 (2016).

[18] C. Cheng, M.-Y. Duan, Z. Wang, and X.-L. Zhou, AlB_2 and MgB_2: a comparative study of their electronic, phonon and superconductivity properties via first principles, Philosophical Magazine **100**, 2275 (2020).

TABLE I. The calculated zone-centre optical phonon frequencies (ν in THz) and their electron-phonon coupling parameters(λ) for hexagonal $LiAlB_4$. IR, R and S indicate infrared-active, Raman-active and silent vibrations, respectively.

Material	E_{1g}(R)	E_{1u}(IR)	E_{1u}(IR)	A_{2u}(IR)	A_{1g}(R)	A_{2u}(IR)	B_{2u}(S)	E_{2u}(S)	B_{1g}(S)	E_{2g}(R)
**LiAlB**$_4$ (ν)	5.719	7.916	10.637	11.020	11.382	12.359	17.382	17.906	18.771	19.999
**LiAlB**$_4$ (λ)	0.669	0.000	0.000	0.000	0.852	0.000	0.000	0.001	0.076	3.551

TABLE II. Structural properties of MgB_2, AlB_2, LiB_2 and $Li_xAl_yB_{2(x+y)}$ (x<6) for this work in bold, and their comparison with previous experimental and theoretical results.

Source	Space group	a(Å)	c(Å)	ΔH (eV/atom)	d_{B-B}(Å)
**MgB_2** (with QE)	**P6/mmm**	**3.074**	**3.508**		**1.775**
**MgB_2** (with CASTEP)	**P6/mmm**	**3.073**	**3.522**		
Exp[5]	P6/mmm	3.083	3.521		
GGA[6]	P6/mmm	3.079	3.515		
GGA[15]	P6/mmm	3.050	3.511		
GGA[16]	P6/mmm	3.051	3.513		
**AlB_2** (with QE)	**P6/mmm**	**3.009**	**3.266**	**-0.523**	**1.737**
Exp[5]	P6/mmm	3.005	3.253		
Exp[17]	P6/mmm	3.004	3.251		
GGA[7]	P6/mmm	3.010	3.240		
GGA[15]	P6/mmm	2.962	3.206		
GGA[16]	P6/mmm	2.983	3.231		
GGA[18]	P6/mmm	3.034	3.226		
**$LiAl_5B_{12}$** (with QE)	**P6/mmm**	**3.008**	**19.882**	**-0.627**	**1.737**
**$LiAl_4B_{10}$** (with QE)	**P6/mmm**	**3.008**	**16.607**	**-0.649**	**1.736**
**$LiAl_3B_8$** (with QE)	**P6/mmm**	**3.006**	**13.349**	**-0.676**	**1.735**
**$LiAl_2B_6$** (with QE)	**$P6_3$/mmc**	**3.005**	**10.095**	**-0.717**	**1.734**
**$LiAlB_4$** (with QE)	**P6/mmm**	**3.000**	**6.705**	**-0.776**	**1.732**
**$LiAlB_4$** (with CASTEP)	**P6/mmm**	**3.000**	**6.691**		
**Li_2AlB_6** (with QE)	**P6/mmm**	**2.991**	**10.239**	**-0.810**	**1.727**
**Li_3AlB_8** (with QE)	**P6/mmm**	**2.987**	**13.539**	**-0.821**	**1.725**
**Li_4AlB_{10}** (with QE)	**P6/mmm**	**2.984**	**16.990**	**-0.807**	**1.723**
**Li_5AlB_{12} (with QE)**	**P6/mmm**	**2.983**	**20.358**	**-0.795**	**1.722**
**LiB_2** (with QE)	**P6/mmm**	**2.971**	**3.455**	**-0.729**	**1.715**
GGA[8]	P6/mmm	2.973	3.466		

TABLE III. The calculated T_c (K) for seven different values of Coulomb parameter (μ^*) in ordered Li-Al-B compounds.

Material	0.10	0.11	0.12	0.13	0.14	0.15	0.16
**$LiAl_3B_8$**	34.66	32.48	30.34	28.24	26.20	24.22	22.29
**$LiAl_2B_6$**	42.87	40.69	38.54	36.42	34.33	32.28	30.26
**$LiAlB_4$**	44.21	41.82	39.50	37.22	34.97	32.77	30.63
**Li_2AlB_6**	75.14	72.50	69.87	67.26	64.65	62.06	59.48
**Li_3AlB_8**	79.42	77.00	74.59	72.18	69.77	67.37	64.98
**Li_4AlB_{10}**	55.38	53.18	50.99	48.82	46.66	44.52	42.40
**Li_5AlB_{12}**	38.93	36.84	34.78	32.77	30.75	28.79	26.884

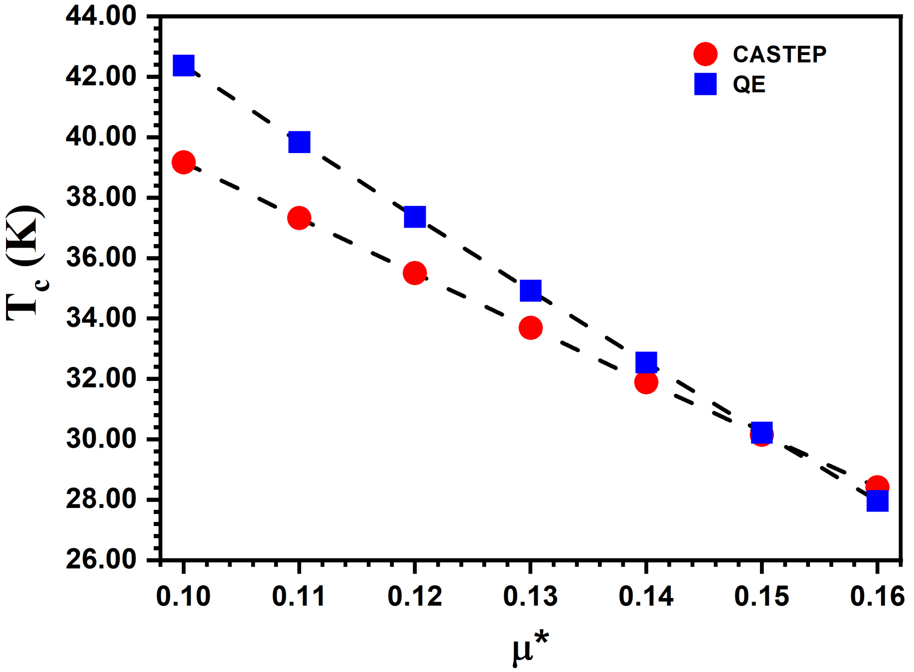

FIG. 1. The change in T_c depending on the choice of μ^* for MgB_2. The CASTEP and Quantum Espresso (QE) results are shown as red circles and blue squares, respectively.

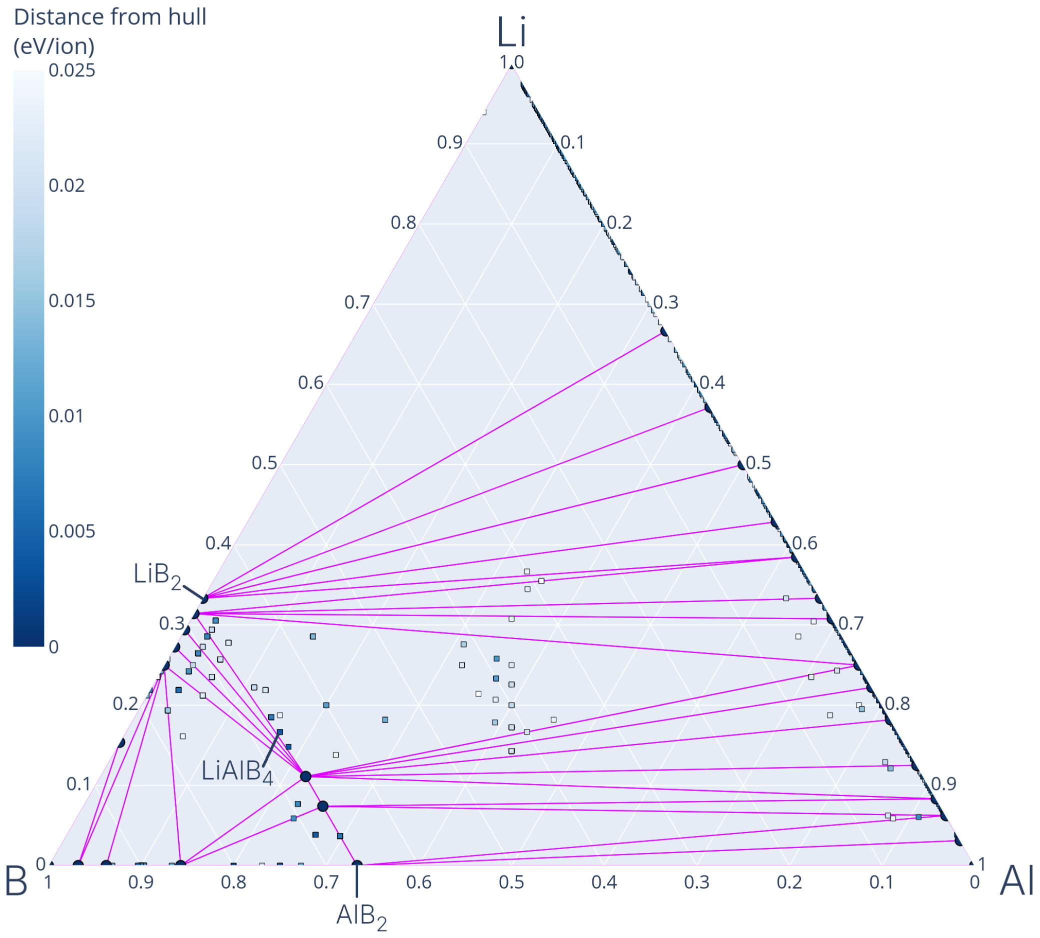

FIG. 2. Structures on and within 0.025 eV/ion of the CHGNet Li-Al-B convex hull. Circles represent structures on the hull, smaller squares represent structures above it and pink lines show the edges of the convex hull. $Li_xAl_yB_{2(x+y)}$ structures lie on a straight line that joins LiB_2 and AlB_2.

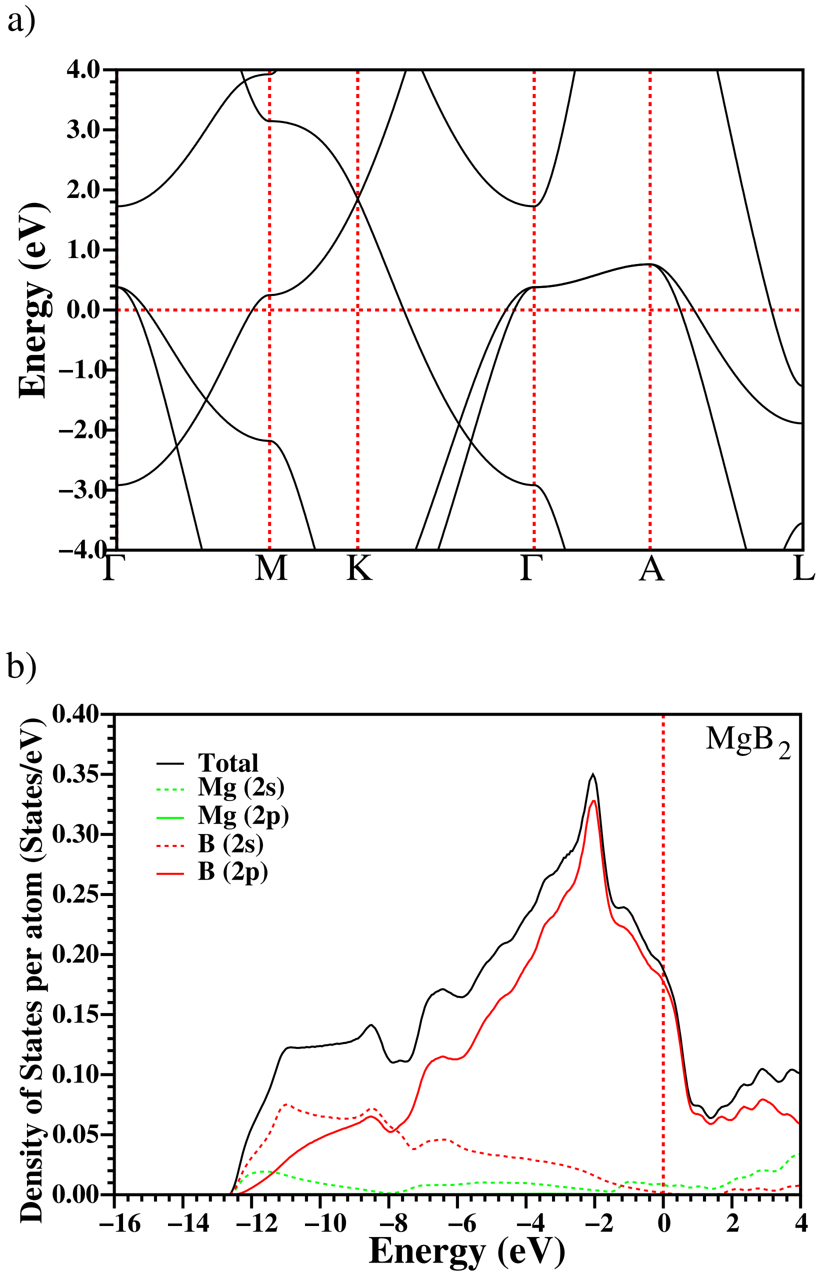

FIG. 3. The calculated electronic band structure along the high symmetry directions in the first Brillouin zone of hexagonal lattice, calculated total and partial electronic density of states for MgB_2

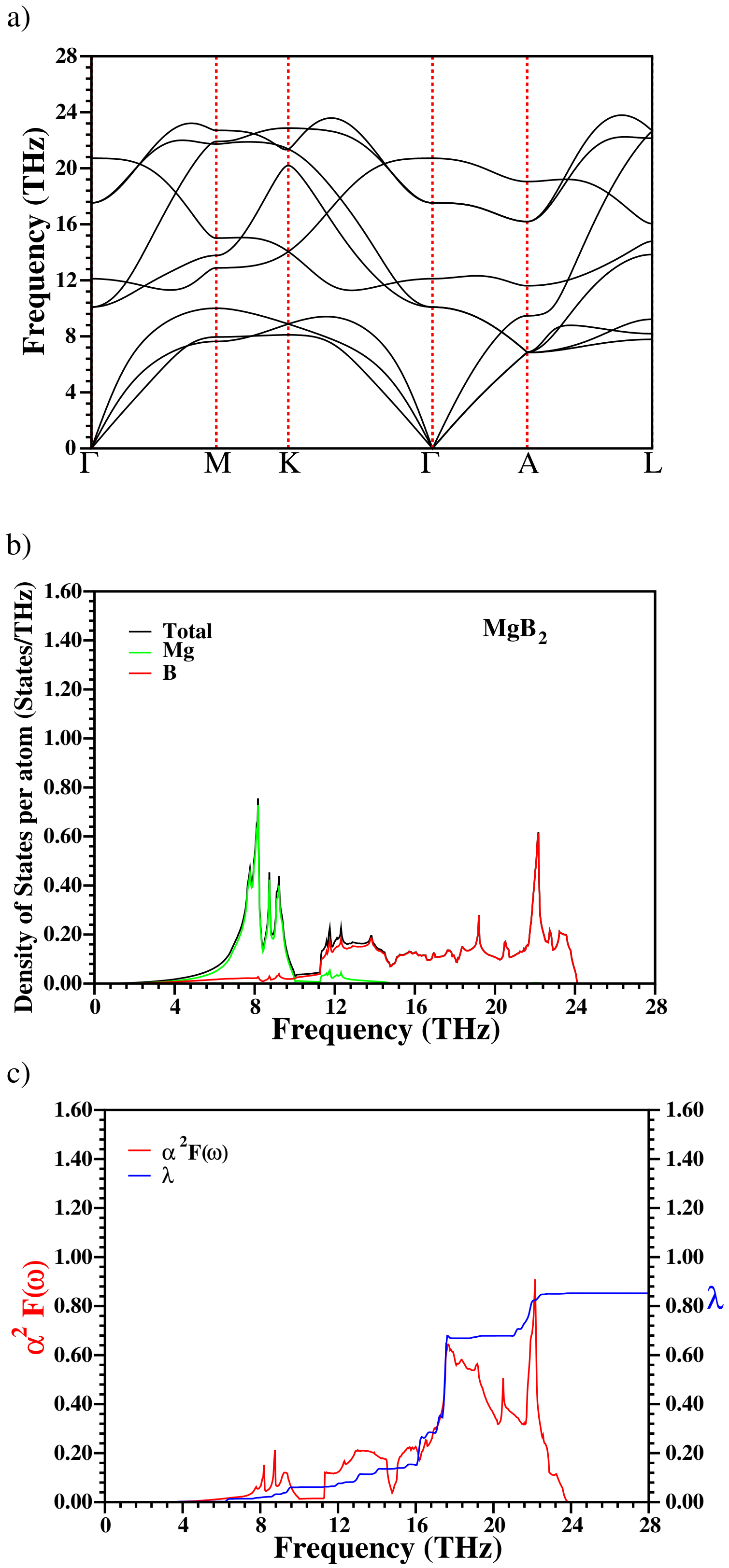

FIG. 4. (a) Phonon dispersion curves, (b) total, partial vibrational density of states and the calculated (c) electron–phonon spectral function $\alpha^2F(\omega)$ (red line) and the variation of the electron–phonon coupling parameter (blue line) with frequency $\lambda(\omega)$ of MgB_2

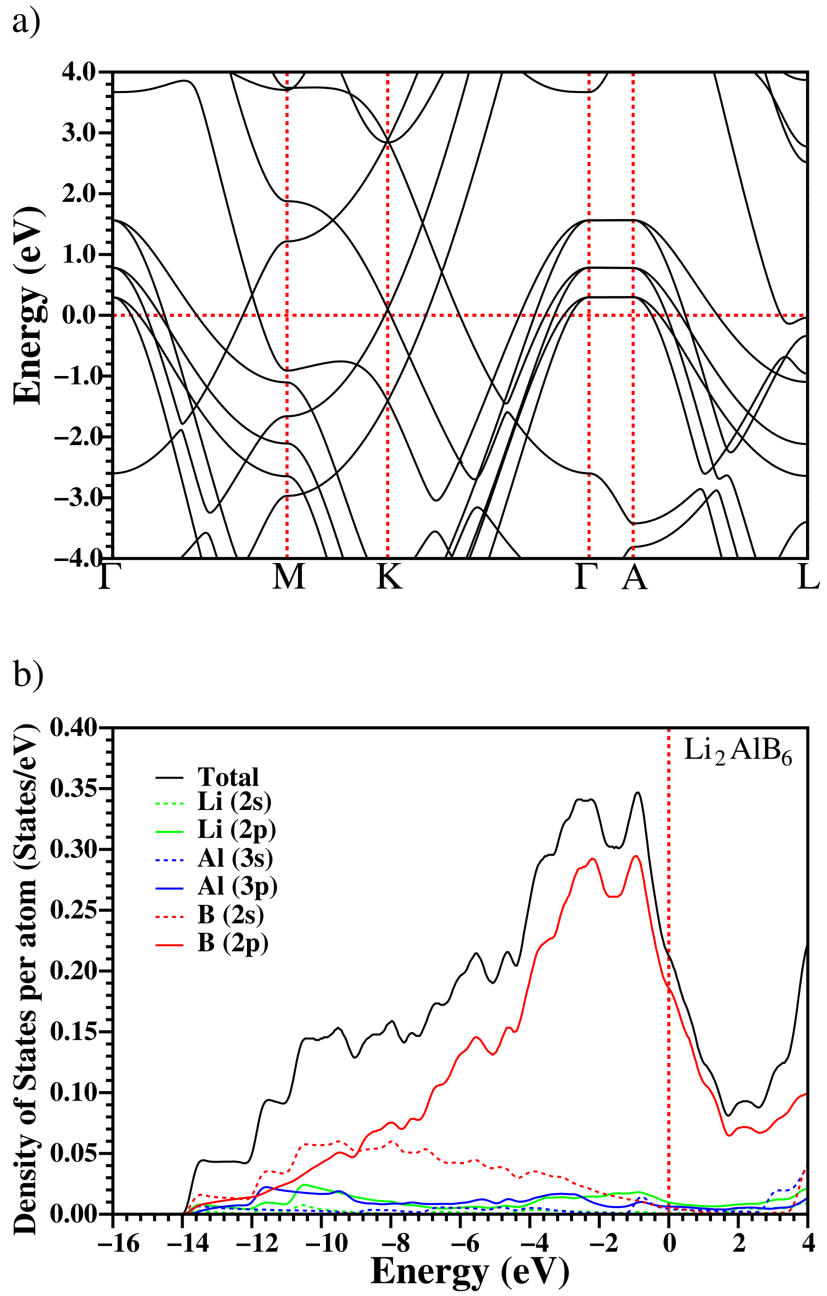

FIG. 5. The calculated electronic band structure along the high symmetry directions in the first Brillouin zone of hexagonal lattice, calculated total and partial electronic density of states for Li_2AlB_6

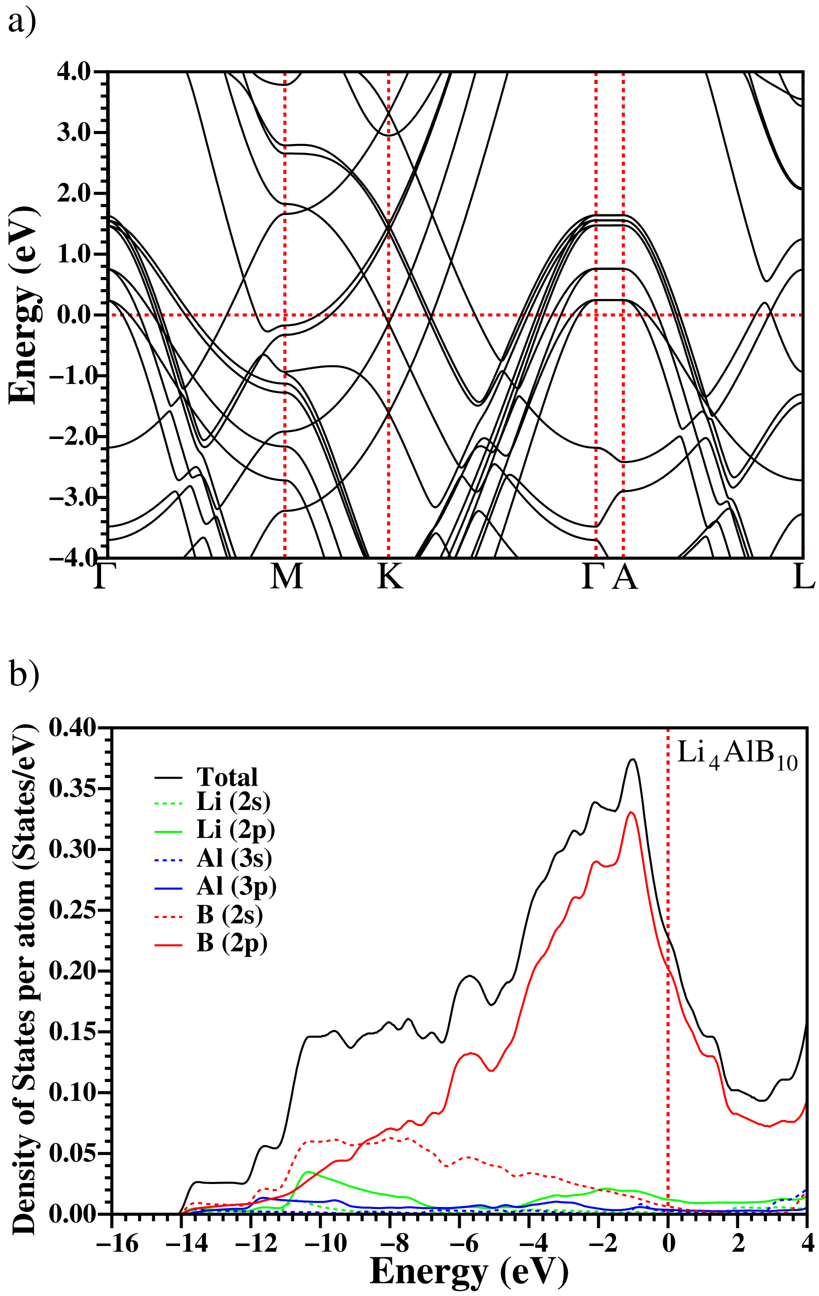

FIG. 6. The calculated electronic band structure along the high symmetry directions in the first Brillouin zone of hexagonal lattice, calculated total and partial electronic density of states for Li_4AlB_{10}

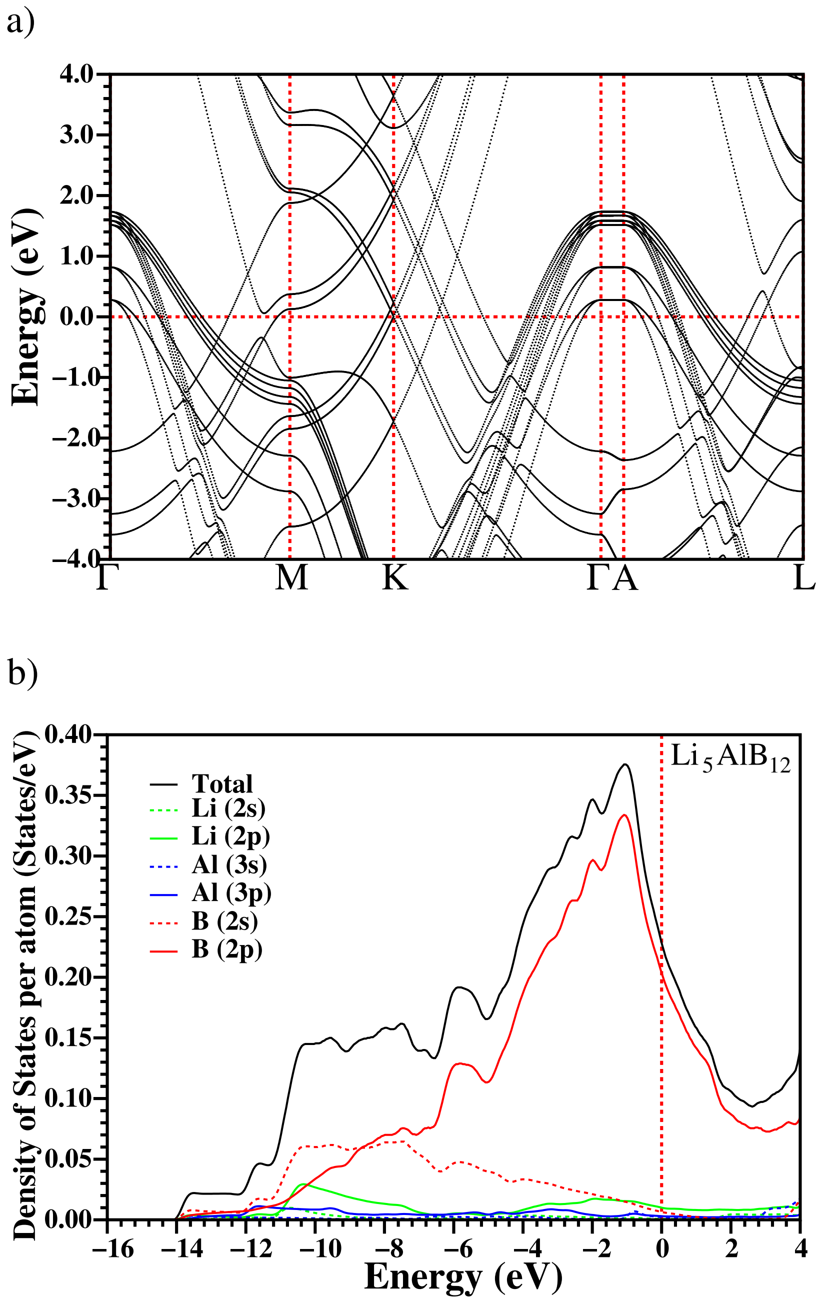

FIG. 7. The calculated electronic band structure along the high symmetry directions in the first Brillouin zone of hexagonal lattice, calculated total and partial electronic density of states for Li_5AlB_{12}

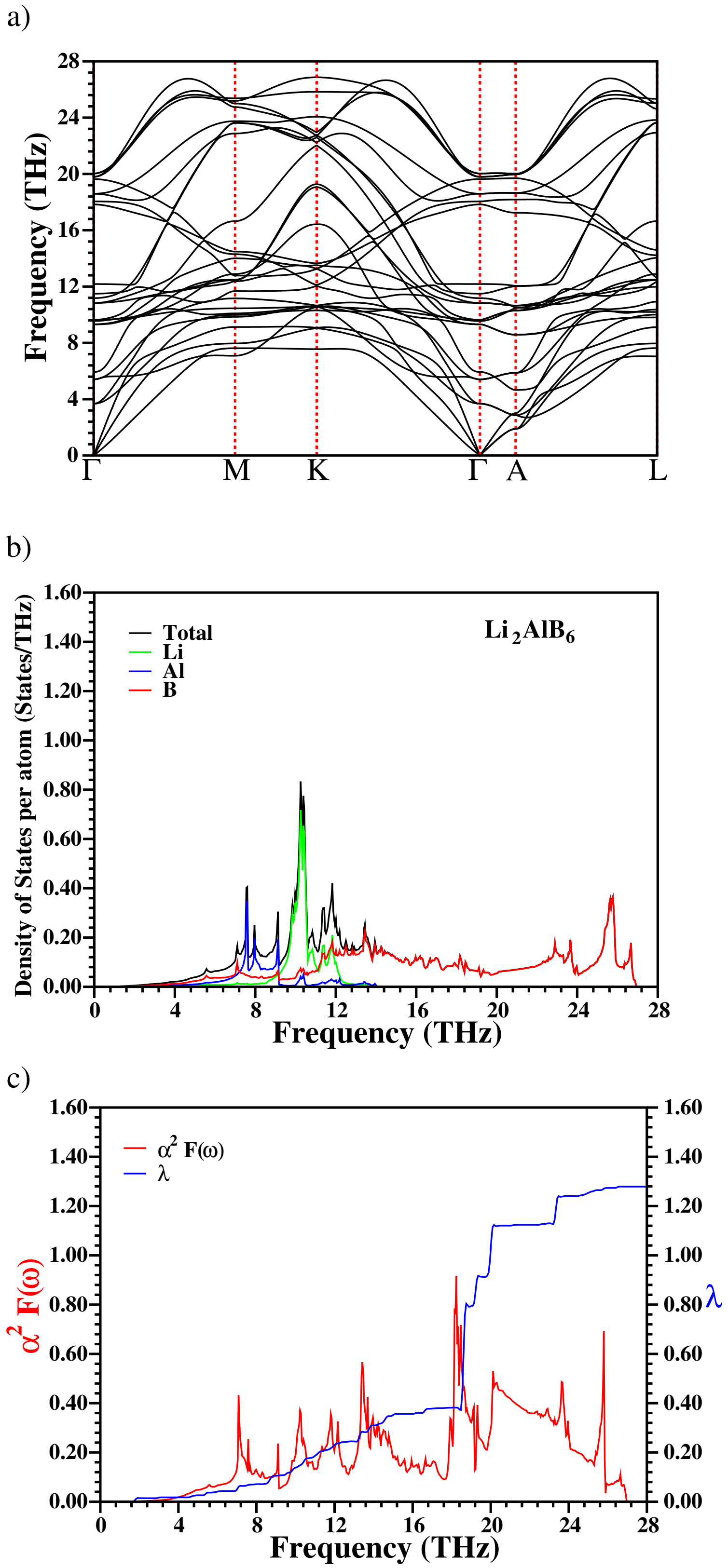

FIG. 8. (a) Phonon dispersion curves, (b) total, partial vibrational density of states and the calculated (c) electron–phonon spectral function $\alpha^2F(\omega)$ (red line) and the variation of the electron–phonon coupling parameter (blue line) with frequency $\lambda(\omega)$ of Li_2AlB_6

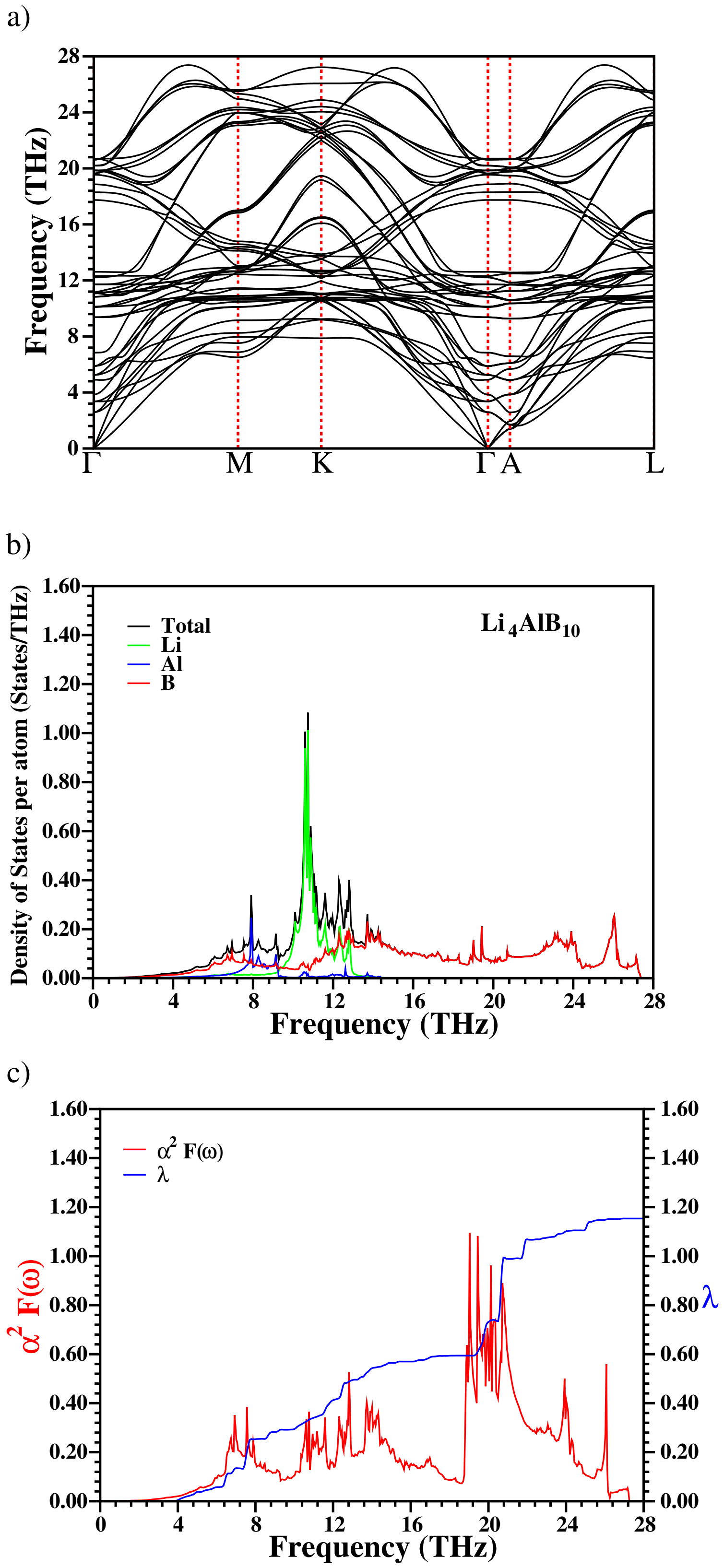

FIG. 9. (a) Phonon dispersion curves, (b) total, partial vibrational density of states and the calculated (c) electron–phonon spectral function $\alpha^2F(\omega)$ (red line) and the variation of the electron–phonon coupling parameter (blue line) with frequency $\lambda(\omega)$ of Li_4AlB_{10}

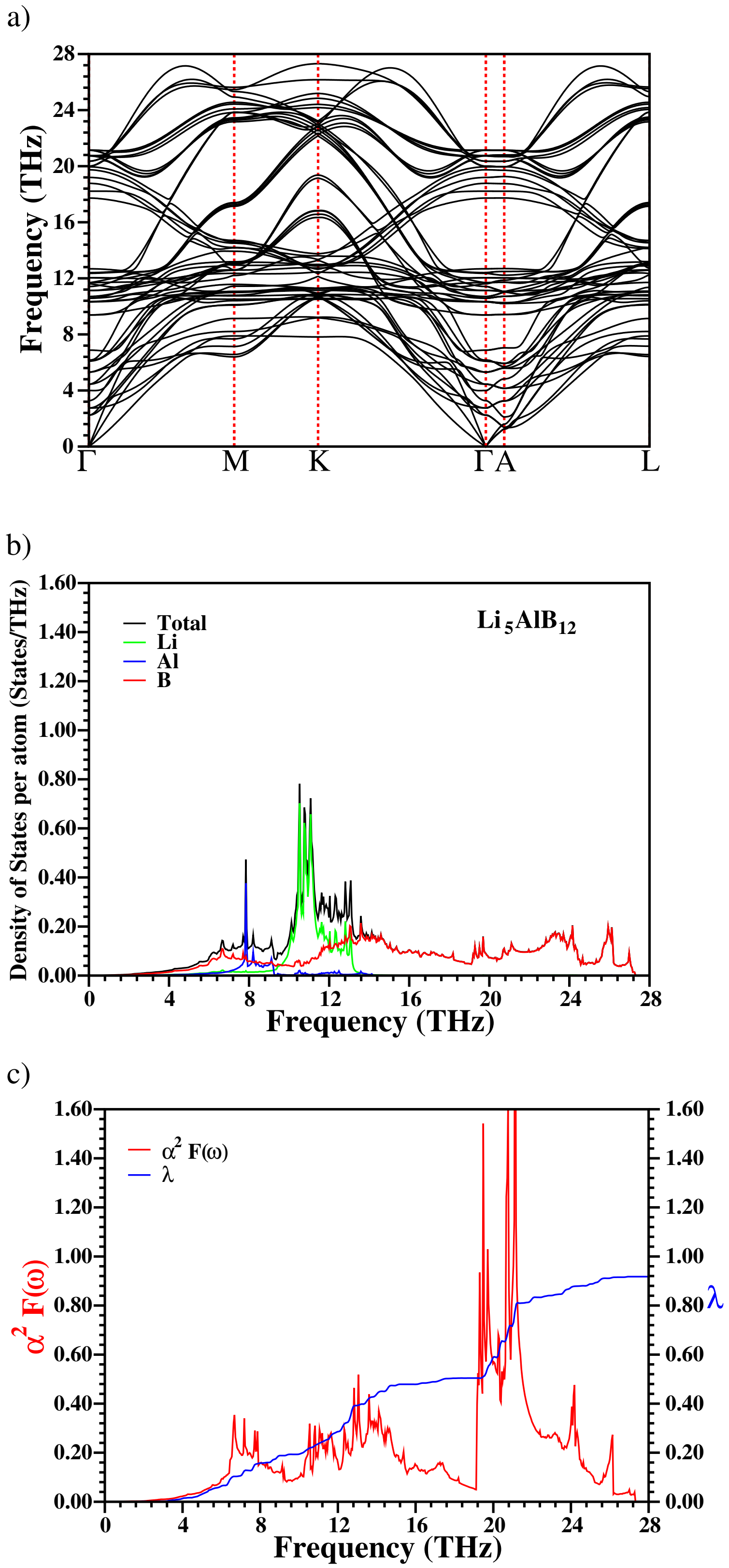

FIG. 10. (a) Phonon dispersion curves, (b) total, partial vibrational density of states and the calculated (c) electron–phonon spectral function $\alpha^2 F(\omega)$ (red line) and the variation of the electron–phonon coupling parameter (blue line) with frequency $\lambda(\omega)$ of Li_5AlB_{12}
